# Sulforaphane Decreases the Burden of AKT1-Expressing Pre-Neoplastic Cells in a Zebrafish Model of Glioblastoma Initiation

**DOI:** 10.3390/nu18162694

**Published:** 2026-08-18

**Authors:** Manana Kutsia, Oliver J. Read, Sharadha Dayalan Naidu, Albena T. Dinkova-Kostova, Dirk Sieger

**Affiliations:** 1 Institute for Neuroscience and Cardiovascular Research, University of Edinburgh, Edinburgh EH8 9YL, UK; dirk.sieger@ed.ac.uk; 2 Cancer Research UK Scotland Centre, Edinburgh EH4 2XR, UK; 3 Department of Physiology, College of Medicine, Korea University, 73 Goryeodae-ro, Seongbuk-gu, Seoul 02841, Republic of Korea; 4 Jacqui Wood Cancer Centre, Division of Cancer Research, Ninewells Hospital and Medical School, University of Dundee, Dundee DD1 9SY, UK; oliver_read@bio-rad.com (O.J.R.); s.z.dayalannaidu@dundee.ac.uk (S.D.N.); a.dinkovakostova@dundee.ac.uk (A.T.D.-K.); 5 Department of Physiology, Pharmacology and Therapeutics, Johns Hopkins University School of Medicine, Baltimore, MD 21205, USA; 6 Department of Medicine, Johns Hopkins University School of Medicine, Baltimore, MD 21205, USA

**Keywords:** AKT1, glioblastoma initiating cells, Nrf2, phytochemicals, sulforaphane, VVD130037

## Abstract

**Background**: Glioblastoma is a primary aggressive brain tumor, with an average survival rate of ~14.6 months. The low therapeutic benefit of current treatments is in part due to glioblastoma-initiating cells hijacking microglia/macrophages to support tumor growth, prompting the development of multitargeted therapeutic approaches. One such therapeutic target is transcription factor Nrf2. High Nrf2 activity is associated with high-grade tumors, and high Nrf2 levels in microglia/macrophages lead to polarization toward an immunosuppressive profile, supporting glioblastoma progression and therapy resistance. Interestingly, however, sulforaphane (SFN), an isothiocyanate found in cruciferous vegetables and a potent Nrf2 activator, has anti-carcinogenic effects in multiple animal models. **Methods**: We utilized the zebrafish glioblastoma initiation model of human AKT1 overexpression in neuronal progenitors to capture the intermediate progenitor-cell-like state of glioblastoma-initiating/pre-neoplastic cells and evaluated the therapeutic potential of SFN and VVD130037, an Nrf2 inhibitor currently in clinical trials. **Results**: Initial findings suggest that SFN, individually and in combination with VVD130037, had the potential to decrease the levels of AKT1. Surprisingly, VVD130037 tended to increase AKT1. The pAKT1 levels also increased in Nrf2-deficient human cells. Moreover, failure to activate Nrf2 in neural progenitors in response to SFN, while a preliminary observation that warrants further investigation, suggests that the decrease in AKT1 was not potentially mediated by Nrf2 activation. The combined effect of SFN and VVD130037 on AKT1 was particularly strong in irf8-/- mutant larvae, which lack a microglia/macrophage population, indicating the existence of a non-tumorigenic cell population(s) sensitive to changes in Nrf2 activity. **Conclusions**: AKT1 inhibition in glioblastoma-initiating cells by the phytochemical SFN, combined with Nrf2 inhibition in the surrounding cells, may impede glioma progression.

## 1. Introduction

Nrf2 is a transcription factor that regulates the expression of gene families encoding proteins with detoxification, antioxidant, and anti-inflammatory functions [1,2]. High Nrf2 activity in various oncogenic pathologies, such as lung, melanoma, and gastric cancer, is associated with therapy resistance, either through supporting the activation of detoxification and anti-apoptotic pathways or establishing Nrf2-mediated immunosuppression [3,4]. Nrf2 is also activated in glioblastoma, where rapid tumor progression and therapeutic resistance are linked to complex interactions between neoplastic cells and tumor-associated microglia/macrophages (TAM) [5,6]. Nrf2’s anti-inflammatory properties become particularly important in the context of microglia/macrophages, which appear to adopt immunosuppressive function and support tumor growth [7,8]. However, one of the most potent naturally occurring Nrf2 activators, sulforaphane (SFN), a dietary compound from cruciferous vegetables, such as broccoli, has demonstrated anti-tumorigenic effects in numerous animal models [9,10,11,12,13,14,15].

Anti-cancer properties of SFN, including inducing apoptosis or decreasing AKT1 and pAKT1 levels, have been documented for glioblastoma, breast, bladder, and prostate cancer [10,11,12,13]. In addition, SFN shows synergistic effects with cancer treatment drugs, suggesting potential beneficial effects to patients by the possibility of using lower drug doses, and there is evidence that SFN increases susceptibility to treatment in drug-resistant cancer cell lines [16]. Notably, SFN has multiple cellular targets, and it is not always clear whether the capacity of SFN to suppress tumors is Nrf2-dependent or independent. Furthermore, considering the potential detrimental effect of high Nrf2 activity in cells comprising the tumor microenvironment, particularly microglia/macrophages, what is the level of SFN (and Nrf2 activation) that is beneficial to achieve a reduction in glioblastoma-initiating cells?

In this study, we addressed these questions using a zebrafish glioblastoma initiation model, which involves overexpression of AKT1 in neural progenitor cells. Our results suggest that AKT1 protein levels increase in response to the Nrf2 inhibitor VVD130037, a compound currently in clinical trials for patients with solid tumors. In agreement, Nrf2-knockout (Nrf2-KO) U2OS human osteosarcoma cells have higher levels of pAKT1, compared to their Nrf2-wild-type (WT) counterparts. These data question the utility of Nrf2 inhibition for the treatment of AKT1-driven glioblastoma and prompted us to conduct experiments with the aim of identifying Nrf2 activity level in a pre-neoplastic system that would exert anti-tumorigenic activity. Interestingly, combining SFN with VVD130037 (which promotes Nrf2 degradation by activating its principal negative regulator, Keap1) [17,18] resulted in AKT1 downregulation, albeit to a smaller extent. Furthermore, in line with previous studies from our group demonstrating low proliferative activity in AKT1-positive (AKT1+) pre-neoplastic cells in the absence of microglia/macrophages, the elimination of microglia/macrophages combined with a dual drug combination of SFN and VVD130037 caused a further decrease in AKT1 levels.

## 2. Materials and Methods

### 2.1. Zebrafish Maintenance

Animal experimentation was approved by the Veterinary Scientific Services of the University of Edinburgh and the Home Office (Project License PP8119722). Zebrafish were housed in a zebrafish facility located at the Queen’s Medical Research Institute, maintained by the University of Edinburgh Bioresearch and Veterinary Services. All zebrafish larvae were kept at 28 °C on a 14-h light/10 h dark cycle. Embryos were obtained from the following adult zebrafish using natural spawning: wildtype (AB) and irf8st95/st95, referred to as irf8-/-, Tg(NBT: DsRed). Embryos were raised at 28.5 °C in E3 medium and treated with 200 μM 1-phenyl 2-thiourea (PTU) starting at the end of 1 dpf for the duration of the experiment.

### 2.2. Cell Culture Maintenance

Nrf2-wild type (WT) and Nrf2-knockout (Nrf2-KO) human osteosarcoma (U2OS) cells were cultured at 37 °C and 5% CO_2_ in Dulbecco’s Modified Eagle Medium (DMEM) containing L-glutamine, sodium pyruvate, and D-glucose (4.5 g/L), and supplemented with 10% (*v*/*v*) fetal bovine serum (FBS) [19]. Human glioma E56 cells were obtained from the Glioma Cellular Genetics Resource (GCGR) and were cultured as described previously [20].

### 2.3. Pharmacological Treatments

Larvae were treated with 10 μM and 40 μM D, L-Sulforaphane (ethanol used as solvent) (Merck, Darmstadt, Germany), 20 μM VVD130037 (0.1% DMSO used as solvent) (TargetMol, Boston, MA, USA), and 40 μM D-Sulforaphane (ethanol used as solvent) (LKT Laboratories, St. Paul, MN, USA), involving a qRT-PCR experiment for FACS-sorted cells. An equal amount of vehicle was added in correspondence to each treatment, and the final solvent concentration was <0.2% (*v*/*v*). To obtain experimental controls, age-matched larvae were treated with a corresponding concentration of Ethanol, which involved either 0.1% or 0.005%. In experiments involving VVD130-037 (TargetMol) larvae, treated in addition with 0.2% DMSO (Sigma, St. Louis, MO, USA), were used as an experimental control. Treatment period indicated in the corresponding figures.

Nrf2-wild type (WT) and Nrf2-knockout (Nrf2-KO) human osteosarcoma (U2OS) cells were seeded into 6-well plates at a density of 3 × 10^5^ per well. On the following day, cells were treated with the NRF2 activator bis(2-hydroxybenzylidene) acetone (HBB2) or vehicle (0.1% DMSO, *v*/*v*) for 18 h [21].

### 2.4. Live Imaging

To label apoptotic cells, embryos were placed in embryo media containing 2.5 μg/mL acridine orange (Sigma-Aldrich, St. Louis, MO, USA) for 30 min. An anesthetic dose of Tricaine (450 μM Tricaine, Sigma-Aldrich) was applied, followed by a wash with embryo media. Embryos were mounted in a 1.5% agarose gel and covered with room temperature embryo media. Both agarose and embryo media contain an anesthetic dose of Tricaine. Imaging was conducted with a Leica SP8 confocal microscope (Mannheim, Wetzlar, Germany) using a 20× air objective (z = 1.3 μm).

### 2.5. Image Analysis

Analyses of all images were conducted using Imaris 8.0.2 (Bitplane, Zurich, Switzerland). For the quantification of RFP+ cells, only cells within the telencephalon and optic tectum were counted for each sample using the ‘Surface’ function tool in Imaris 8.0.2. The same strategy was applied to create acridine orange-positive surfaces. To quantify the apoptosis level in RFP+ cells, the total volume of RFP cells positive for acridine orange signal (confirmed by colocalization of RFP+ and acridine orange+ surfaces) was divided by the total volume of RFP cells of the respective sample. Quantifications were plotted as the percentage of RFP cells positive for acridine orange signal.

### 2.6. RNA Isolation and cDNA Synthesis

Zebrafish cell suspension, including microglia labelled with the 4c4 antibody, for FACS sorting was obtained from 4dpf heads of Tg(NBT: RsRed) treated with 40 μM D-Sulforaphane starting at 3 dpf, according to a previously published protocol [22]. All experiments were conducted with at least two biological replicates. Following sorting of the target cell population, RNA was isolated using the Qiagen RNeasy Plus Micro kit according to the manufacturer’s guidance. RNA samples with RIN score > 7 were used for cDNA synthesis and downstream analysis. cDNA was synthesized using the Super-Script^®^ III First-Strand Synthesis System (Invitrogen, Carlsbad, CA, USA)according to the manufacturer’s guidelines.

For gene expression analysis of E56 cells treated with VVD130037, cells were seeded on laminin-coated 6-well plates (2 µg/cm^2^—R&D Systems, Minneapolis, MN, USA) @ 400,000 cells/well and allowed to adhere for 24 h. Cells were then treated with either 100/500 nM of VVD-130037 or 0.1% DMSO for 18 h, after which wells were washed with ice-cold PBS and lysed for RNA. mRNA was purified using a PureLink RNA Mini Kit (Thermo, Waltham, MA, USA), and the concentration was determined by NanoDrop. Reverse transcription was then performed using PrimeScript RT Master Mix (Takara, Kusatsu, Japan) to generate template cDNA.

### 2.7. Quantitative PCR

Quantitative PCR (qPCR) amplifications were performed in triplicate in a 10 μL reaction volume containing SsoAdvanced Universal SYBR Green Supermix (Bio-Rad, Hercules, CA, USA) using an ABI Quantstudio 96-well system (Thermofisher, Waltham, MA, USA). The PCR protocol used was an initial denaturation step of 10 min at 95 °C, and 45 cycles of 15 s at 95 °C, 1 m at 60 °C, and 15 s at 95 °C. Primers used were as follows:

Beta-actin forward 5′-CACTGAGGCTCCCCTGAATCCC-3′

Beta-actin reverse 5′-CGTACAGAGAGAGCACAGCCTGG-3

gclc forward 5′-TTCCGTTGTCGAAGGTGGATGA-3′

gclc reverse 5′-CCAGTCCATTCTGAGCTGCG-3′

qPCR samples were prepared in PowerUP SYBR Green Master Mix (Thermo), and analysis of gene expression was performed using a QuantStudio 7 Pro Real-Time PCR System (Applied Biosystems, Waltham, MA, USA). Primers used (human-targeting) were as follows:

NFE2L2 forward 5′-TCAGCGACGGAAAGAGTATGA-3′

NFE2L2 reverse 5′-CCACTGGTTTCTGACTGGATGT-3′

NQO1 forward 5′-ATGGTCGGCAGAAGAGC-3′

NQO1 reverse 5′-GGAAATGATGGGATTGAAGT-3′

HMOX1 forward 5′-TCTCCGATGGGTCCTTACACTC-3′

HMOX1 reverse 5′-GGCATAAAGCCCTACAGCAACT-3′

GCLM forward 5′-ACTCTTCATCATCAACTAGAAGTGC-3′

GCLM reverse 5′-CTAATTCCTCCCAGTAAGGCTGT-3′

KEAP1 forward 5′-CAATGAACACCATCCGAAGCG-3′

KEAP1 reverse 5′-CCATCATAGCCTCCAAGGACG-3′

GUSB forward 5′-CGTGGTTGGAGAGCTCATTTGGAA-3′

GUSB reverse 5′-ATTCCCCAGCACTCTCGTCGGT-3′

GAPDH forward 5′-CAACGGATTTGGTCGTATTGGGC-3′

GAPDH reverse 5′-CGCTCCTGGAAGATGGTGATG-3′

### 2.8. Western Blot Analysis

Zebrafish protein lysate, either from whole larvae or larval heads (indicated in figure legends), was prepared using the following steps. Zebrafish larvae with the Irf8-/- mutation are characterized by lower life expectancy, coupled with lower breeding rates and fragile larvae. Furthermore, the combination of high cost and high amounts of each compound required for each treatment condition prompted us to limit Western blot experiments to two biological replicates in each treatment combination. To offset this limitation, all experiments were conducted with embryos of mixed genetic background, and protein samples were pooled from a minimum of *n* = 7 zebrafish embryos. 8dpf larval heads or whole bodies were collected in ice-cold Media A (15 mM Hepes (Gibco, Waltham, MA, USA) and 25 mM D-Glucose (Sigma-Aldrich) in HBSS (Gibco) and homogenized using a glass homogenizer. The cell suspension was strained through a 40-micron filter, followed by centrifugation at 300× *g* at 4 °C for 10 min to pellet the cells. Supernatant was removed, and RIPA (Sigma-Aldrich) +PI (Protease Inhibitor) (Thermofisher) solution was added. The next step involved vortexing and centrifuging the solution at 14,000 rpm and 4 °C for 15 min. The supernatant was collected for further experiments. Protein concentration was calculated using the BCA assay (Thermo Scientific™). We loaded equal amounts of protein based on BCA calculations and normalized (Proteins were reduced 5% *v*/*v* 2-mercaptoethanol and boiled at 60 °C for 30 min). Following the calculation of protein concentration, the same amount of total protein was loaded for each experimental group. Proteins were separated by TGX pre-cast gels (Bio-Rad) and transferred to PVDF membrane (Bio-Rad). Primary antibody(ant-AKT1(Invitrogen)) incubation was done overnight at 4 °C, followed by secondary antibody (Peroxidase AffiniPure Anti-Rabbit IgG (H + L) (Jackson ImmunoResearch, West Grove, PA, USA)) incubation for 2 h. Analysis was conducted using the ImageJ (1.54g, 64-bit Java 8 for Windows) gel analysis function. Due to difficulty identifying the loading control antibody that showed a clear signal with small amounts of protein, the treatment group’s signal was normalized to control treatment groups to evaluate changes in protein expression level.

Following treatment, U2OS cells were lysed in 150 μL of non-reducing sample buffer [50 mMTris–HCl pH 6.8, 2% (*w*/*v*) SDS, 10% (*v*/*v*) glycerol, and 0.02% (*w*/*v*) bromophenol blue]. Whole-cell lysates were boiled at 100 °C for 5 min, sonicated, and protein concentrations determined using the BCA assay (Thermo). Proteins were reduced with 6% (*v*/*v*) 2-mercaptoethanol, resolved using 4–12% NuPAGE, transferred to 0.45 μm nitrocellulose membranes, and subjected to immunoblotting.

## 3. Results

### 3.1. Administration of Sulforaphane as a Single Agent Leads to a Decrease in AKT1-Expressing Pre-Neoplastic/Glioblastoma-Initiating Cells

**Figure 1:**
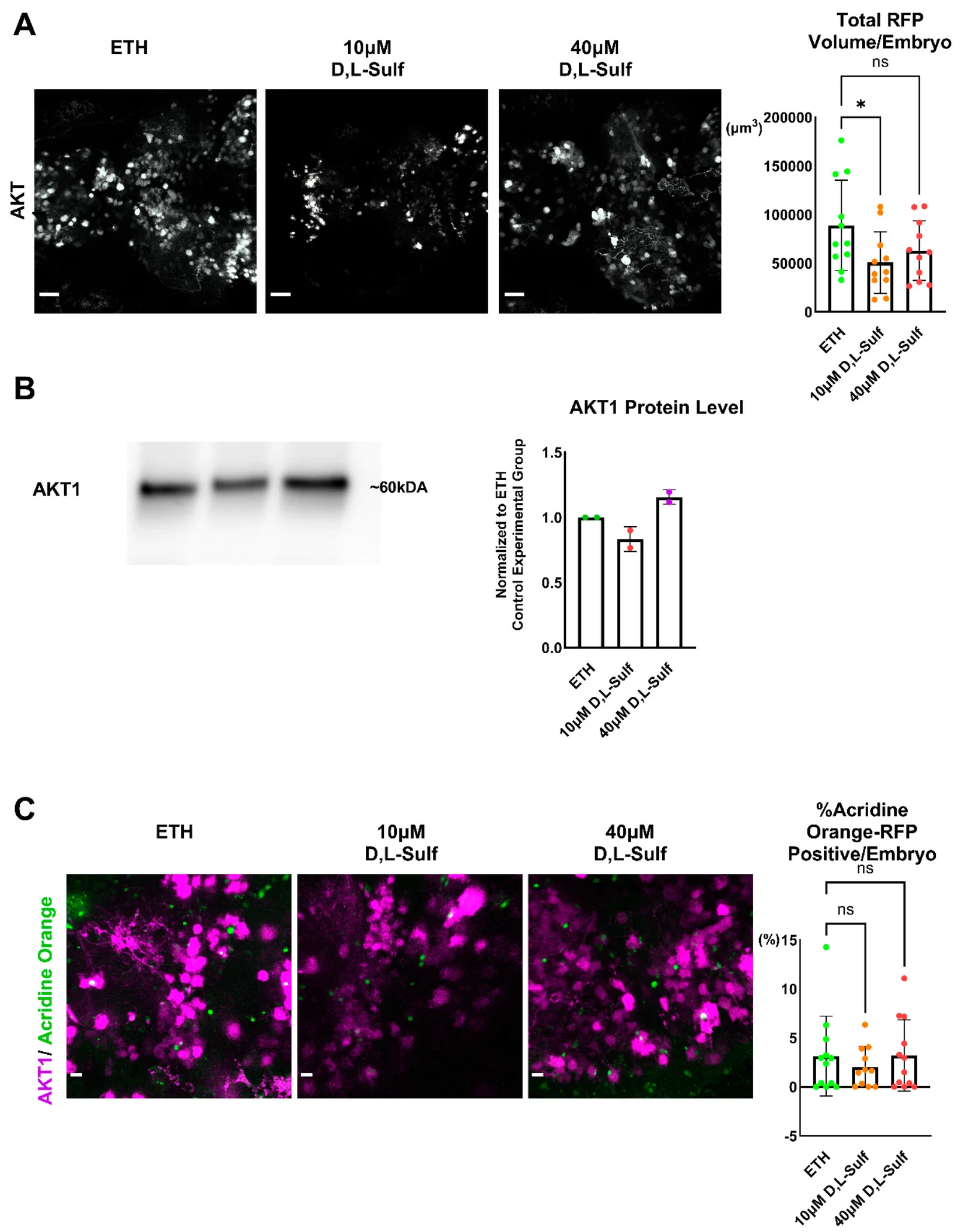
(**A**) Representative images of 8dpf zebrafish larvae co-injected with a driver plasmid containing the NBT promoter (NBT:∆lexPR-lexOP-pA) and a lexOP: AKT1-lexOP: tagRFP, treated with Sulforaphane for 96 h starting at 4dpf (pre-neoplastic/GICs overexpressing AKT1 labelled by the RFP fluorescent protein represented in white color in images). Corresponding quantification of total RFP volume in the telencephalic and optic tectum regions (Control/Eth: 88,997 μm^3^, *n* = 11 larvae; 10 μM D, L-Sulforaphane 50,685 μm^3^, *n* = 11 larvae; 40 μM D, L-Sulforaphane 62,975 μm^3^, *n* = 11 larvae; *N* = 3. Statistical analysis used One-Way ANOVA followed by Dunnett’s post-hoc test; the mean of each treatment group was compared to the mean of the control/Eth treated group. * *p* < 0.05. Error bars represent means ± SD. (**B**) AKT1 protein analysis by Western blot. Protein was isolated from whole zebrafish larvae (To pool protein samples a minimum number of *n* = 7 zebrafish embryos were used). Corresponding expression level analysis using the ImageJ gel band quantification function (*N* = 2). (**C**) Representative images of 8dpf zebrafish larvae co-injected with a driver plasmid containing the NBT promoter (NBT:∆lexPR-lexOP-pA) and a lexOP:AKT1-lexOP:tagRFP, treated with Sulforaphane (AKT1 overexpressing pre-neoplastic/GIC represented in purple, acridine orange labelling represented in green). Corresponding quantification of the percentage of apoptotic RFP pre-neoplastic cells in the telencephalic and optic tectum regions (Control/Eth: 3.144% *n* = 12 larvae; 10 μM D, L-Sulforaphane 2.037% *n* = 11 larvae; 40 μM D, L-Sulforaphane 3.209% *n* = 12 larvae; *N* = 3. Statistical analysis used One-Way ANOVA; the mean of each treatment group was compared to the mean of the control/Eth treated group. Error bars represent mean ± SD.

An in vivo study of ectopic GBM10 xenografts in mice showed a significant reduction in tumor size in response to SFN treatment [10]. To emulate this experiment, we employed our previously published zebrafish glioblastoma initiation model, which is based on co-expression of AKT1 and RFP under the control of a neural-specific beta-tubulin (NBT) promoter using a dominant-active version of the LexPR transcriptional activator system (DLexPR) [23]. Following treatment with 10 or 40 μM D, L-sulforaphane (SFN), to assess the potential anti-tumorigenic activity, we quantified the total volume of AKT1-RFP in response to treatment. To quantify apoptotic AKT1+ preneoplastic/GICs, we used acridine orange labeling. Interestingly, we observed a decreased AKT1+ burden at lower concentrations of SFN (Figure 1A). This effect was lost in the 40 µM SFN-treated group, and we observed only a trend towards a minor decrease in total AKT1 pre-neoplastic volume (Figure 1A).

We confirmed these results by protein isolation from the whole zebrafish body and quantifying the protein levels of AKT1 in response to SFN treatment (Figure 1B). In line with the immunohistochemistry data, we observed a subtle decrease in AKT1 protein in response to the lower concentration of SFN, while the opposite was true for the higher 40 µM SFN treatment. These data highlight the importance of tightly regulating the concentration of SFN to achieve beneficial effects.

To assess whether the reduction in pre-neoplastic mass was due to SFN affecting the survival of these cells, we quantified the total volume of RFP surfaces positive for acridine orange and normalized it by the total volume of RFP in the respective sample. We did not detect any noticeable change in the percentage of apoptotic glioblastoma-initiating cells (GICs) following SFN treatment compared with vehicle treatment (Figure 1C). These results suggest a non-apoptosis-mediated decrease in AKT1 levels by SFN.

### 3.2. Sulforaphane Decreases AKT1 in Neuronal Progenitors in an Nrf2-Independent Manner

**Figure 2:**
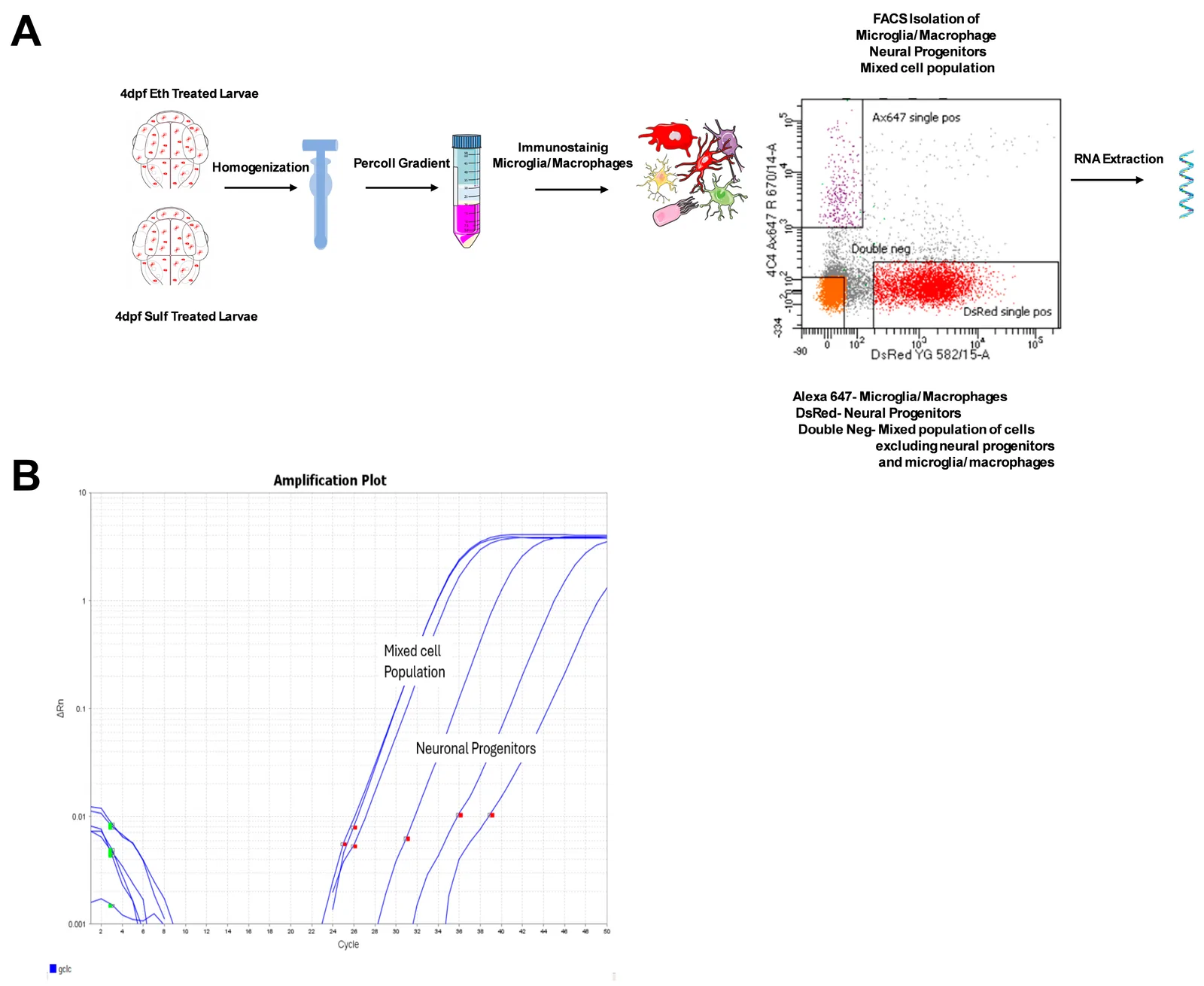
(**A**) Schematic representation of isolating target cell populations for mRNA extraction, adapted from [19]. (**B**) RT-PCR amplification plot comparison for *gclc* in a mixed population of cells and neural progenitors.

Our next goal was to elucidate whether the observed decrease in AKT1 protein level upon SFN treatment in neuronal cells was mediated by Nrf2 activation. A previous publication has reported the epigenetic repression of Nrf2 in murine developmental neurons, which ensures the maturation of these cells [24]. These cells not only have an overall low basal level of Nrf2 target genes but Nrf2 inducers or Keap1 ablation also fail to upregulate Nrf2 activity [24]. We wanted to confirm if similar phenomena exist in zebrafish neuronal cells. For this purpose, we treated the Tg(NBT: DsRed) larvae, which label neuronal cells with 40 μM SFN for 16 h starting at 3dpf, and using a previously published FACS sorting protocol [22], we isolated mRNA from the following three cell populations: microglia/macrophages, neuronal progenitors, and a mixed population of cells that do not contain either microglia/macrophages or neurons (Figure 2A). To evaluate Nrf2 activity in these cells in response to SFN treatment, we quantified the relative expression level of *gclc*, a classical Nrf2 target gene. In line with previous reports, we saw a very low level of *gclc* expression in control and SFN-treated neuronal progenitors, characterized by high and variable Ct values (Figure 2B).

**Figure 3:**
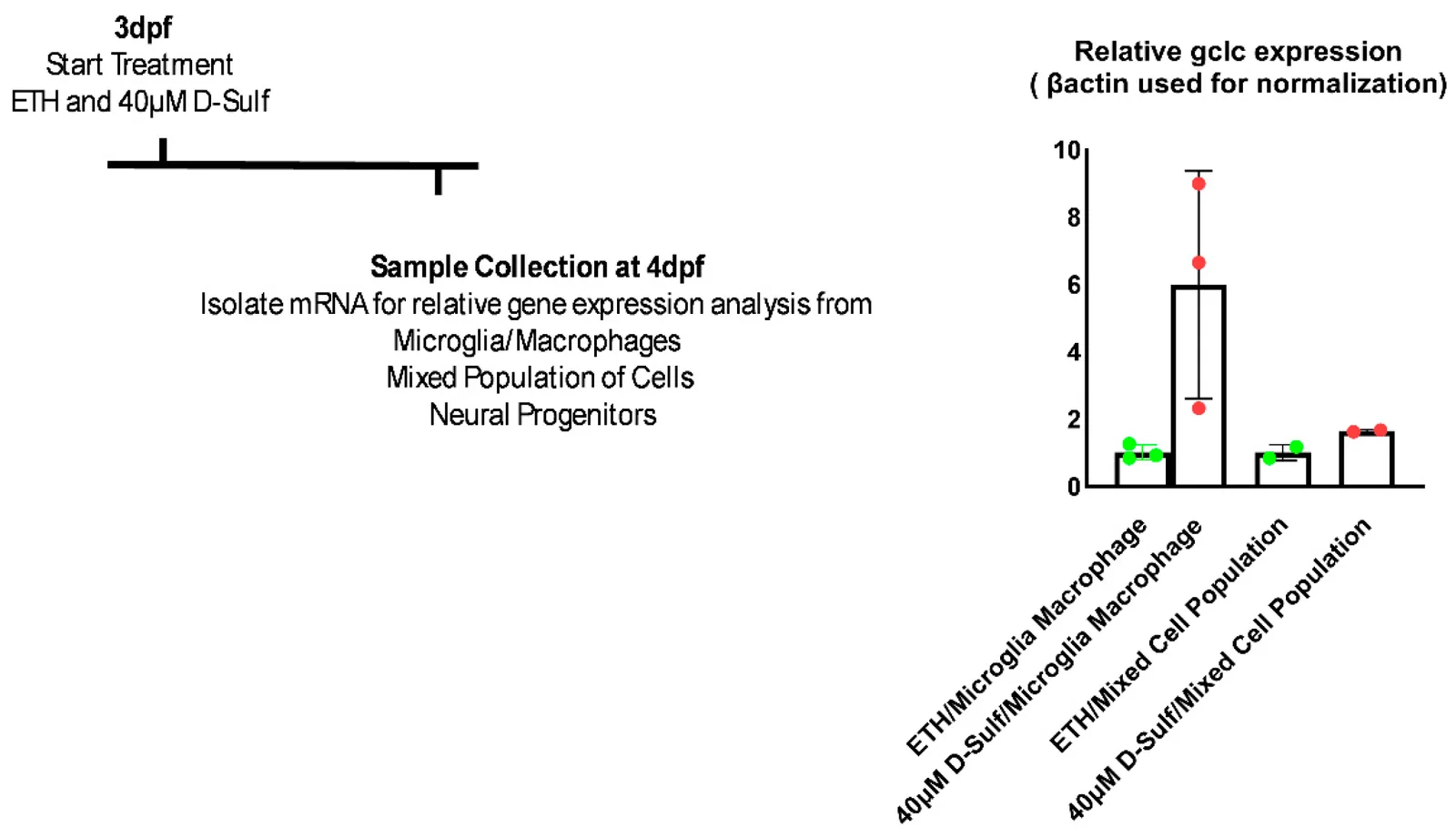
Treatment and sample collection timeline for conducting Nrf2 target gene expression analysis in the target cell population. Changes in mRNA expression levels of gclc in microglia/macrophages (*N* = 3) and a mixed population of cells (*N* = 2) in response to 16 h 40 µM Sulforaphane treatment (For each FACS sample, a minimum of *n* = 100 zebrafish embryo heads were pooled). Fold change calculated using the (ΔΔCT) method. Error bars represent means ± SD.

In comparison, we detected a clear increase in *gclc* mRNA levels in microglia/macrophages, while the mixed population of cells showed a subtle increase (Figure 3). These results imply a potentially Nrf2-independent mechanism of AKT1 downregulation in neuronal progenitors after SFN administration.

### 3.3. Combination Treatment with Sulforaphane and the Nrf2 Inhibitor VVD130-037 Lowers the Levels of AKT1

**Figure 4:**
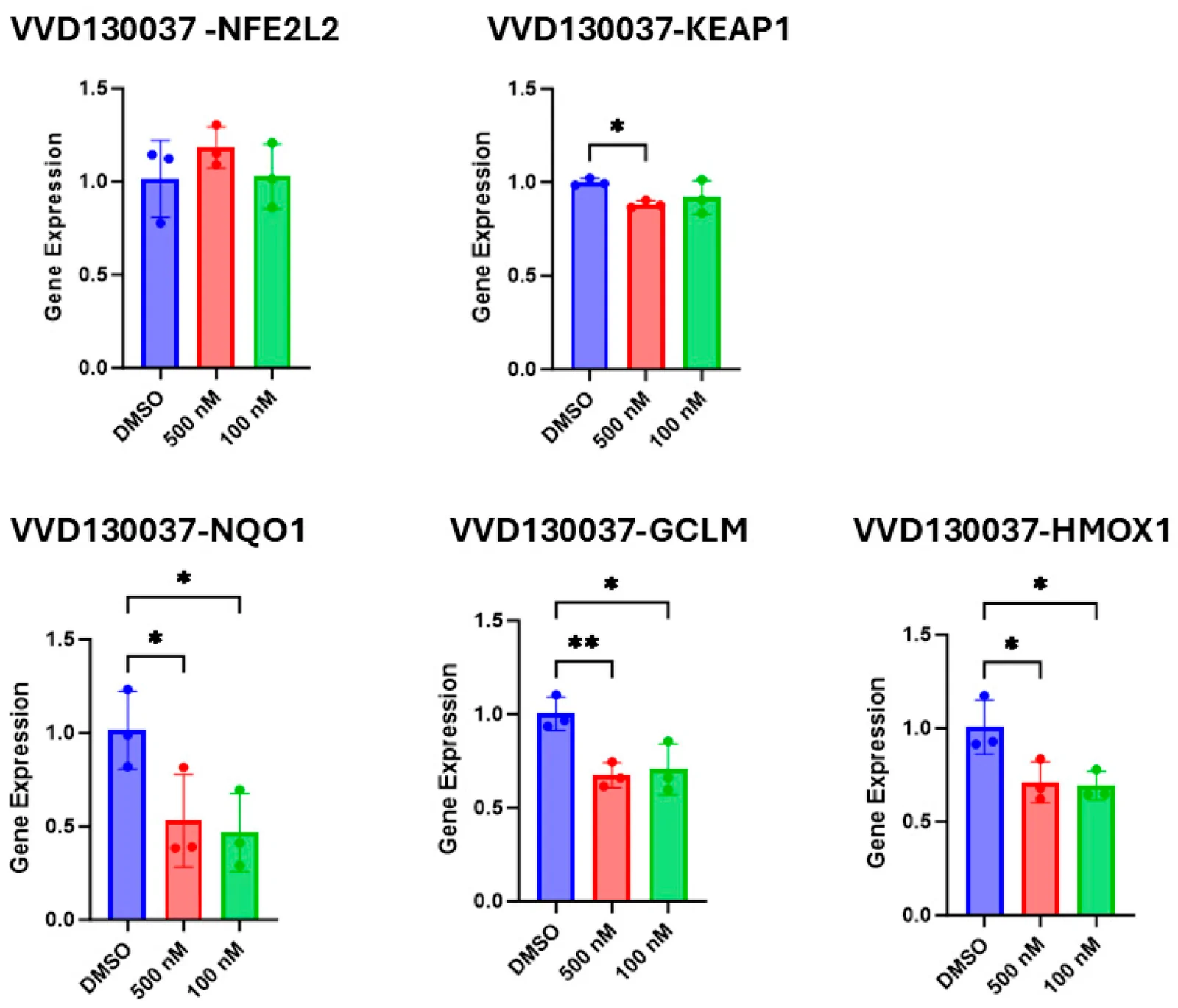
mRNA expression analysis in response to the NRF2 inhibitor VVD130-037. Human glioma cells (400,000 cells/well) were seeded on laminin-coated 6-well plates and allowed to adhere for 24 h. Cells were then treated with vehicle (0.1% DMSO) or VVD130-037 at the indicated concentrations. The mRNA levels for Nrf2 (gene name *NFE2L2*), *KEAP1*, *NQO1*, *GCLM*, and *HMOX1* were determined 18 h post-treatment. Statistical analysis used One-Way ANOVA followed by Dunnett’s post-hoc test, with the mean of each treatment group compared to the mean of the DMSO-treated group. * *p* < 0.05 and ** *p* < 0.01. Error bars represent means ± SD.

Intrigued by the absence of inducibility of Nrf2 in neural progenitors upon SFN treatment and the decrease in total volume of GICs overexpressing AKT1 in response to 10 μM SFN, we hypothesized that we could further decrease the AKT1 levels if we controlled for the upregulation of Nrf2 by SFN in the cells surrounding the AKT1-overexpressing cells. Our rationale was that combining the Nrf2 inhibitor VVD130037 [17,18] with SFN would allow SFN to exert Nrf2-independent anti-tumorigenic effects on malignant neuronal progenitors without altering basal Nrf2 levels in other cell populations, which could otherwise create a pro-tumorigenic environment. To further confirm our hypothesis regarding the importance of maintaining Nrf2 at a basal level, in addition to the dual treatment with VVD130037/SFN, we also included an experimental group treated solely with VVD130037, thereby decreasing Nrf2 levels compared to the steady state. To confirm the Nrf2 inhibitory effect of VVD130037, we treated human glioblastoma cells (E56, obtained from the Glioma Cellular Genetics Resource, GCGR, University of Edinburgh) with 100 or 500 nM of VVD130037. We measured the mRNA levels for the Nrf2 target genes NQO1, GCLM, and HMOX1. Indeed, we observed downregulation of all Nrf2 target genes, although there were no detectable differences between 100 and 500 nM VVD130037 treatment, confirming that this compound is an inhibitor of Nrf2-dependent transcription (Figure 4).

**Figure 5:**
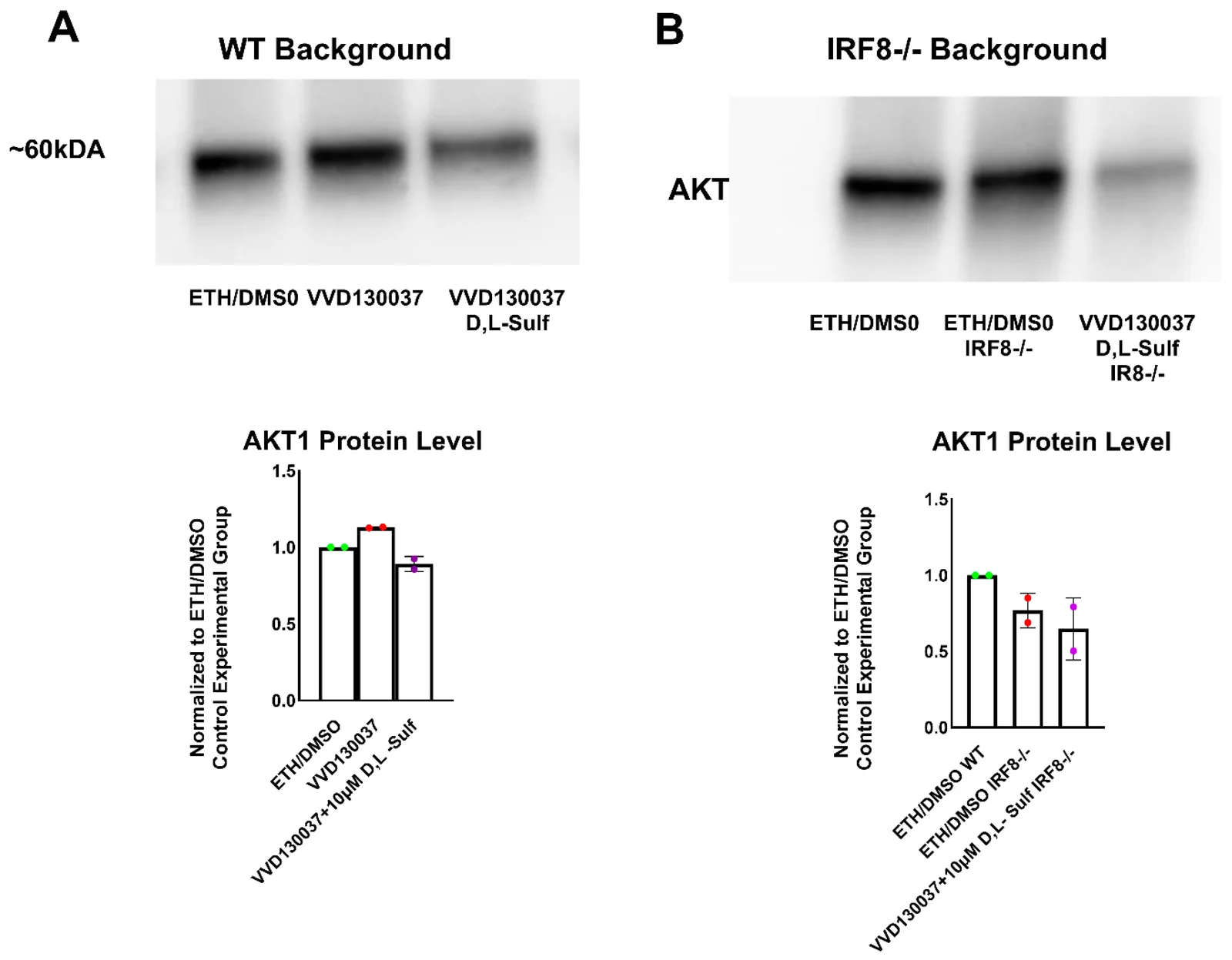
(**A**) AKT1 protein analysis by Western blot. Protein was isolated from whole zebrafish larvae. Each group was treated with a corresponding compound combination starting at 4dpf for 96 h. Protein samples were collected at 8dpf. Corresponding expression level analysis using the ImageJ gel band quantification function (*N* = 2) (to pool protein samples, a minimum of *n* = 7 zebrafish embryos were used). (**B**) AKT1 protein analysis by western blot. Protein is isolated from the zebrafish head only with the IRF8-/- background. Corresponding expression level analysis using the ImageJ gel band quantification function (*N* = 2) (to pool protein samples, a minimum of *n* = 7 zebrafish embryos were used). Error bars represent means ± SD.

To validate our hypothesis regarding the importance of maintaining basal Nrf2 expression in non-AKT1 cells, we collected protein samples from zebrafish embryos overexpressing AKT1 from the following experimental groups: ETH/DMSO vehicle-treated group, 20 μM VVD130-037-treated group, and 20 μM VVD130037/10 μM SFN combination-treated group. Indeed, in the VVD130037 treatment group, we observed a nominal increase in AKT1 levels (Figure 5A). Although further investigation is required to confirm VVD130037-mediated Nrf2 downregulation under these experimental conditions, considering the known high Keap1/Nrf2 selectivity of this compound [17], these preliminary data highlight the importance of maintaining Nrf2 at a basal level of expression even under pre-neoplastic conditions.

**Figure 6:**
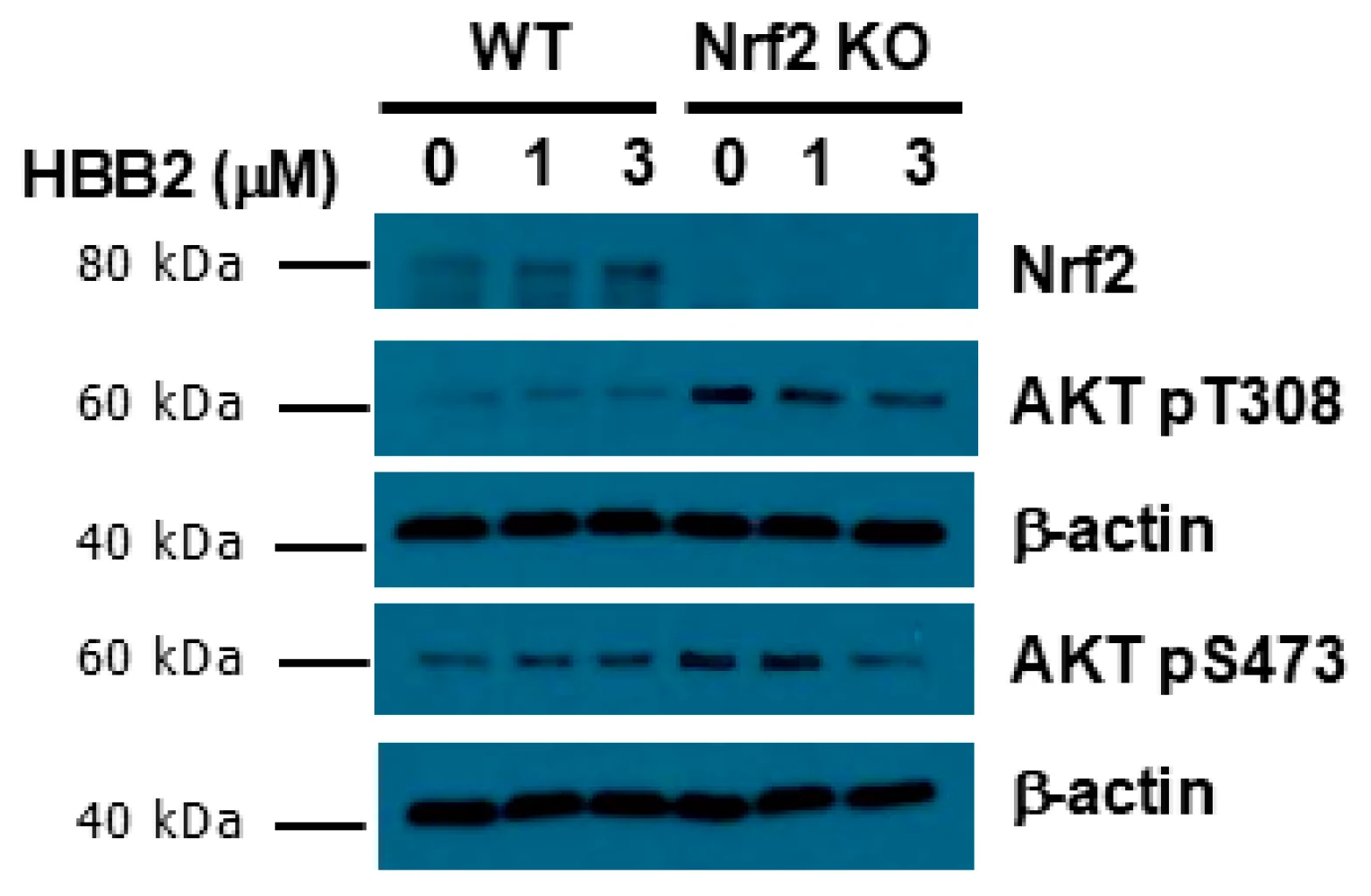
U2OS cells and their counterparts in which Nrf2 has been deleted using CRISPR/Cas technology were treated with the Nrf2 activator bis(2-hydroxybenzylidene)acetone (HBB2) for 18 h, and the protein levels of Nrf2 and AKT phosphorylated at either T308 or S473 were determined by immunoblotting.

Further evidence for the importance of Nrf2 in keeping low AKT1 levels was obtained from an experiment comparing the levels of phosphorylated (active) AKT1 in human U2OS cells and their Nrf2-knockout counterparts [19]. In this system, the levels of phosphorylated AKT at both Thr308 and Ser473 were increased in Nrf2-deficient cells, and the effect of pAKT1 upregulation was diminished by administration of bis(2-hydroxybenzylidene) acetone (HBB2) (Figure 6). Further validation is required to determine whether a similar mechanism of AKT1 and pAKT1 downregulation exists in glioblastoma cell lines.

Notably, this experiment also revealed that, as with SFN, the effect of HBB2, a potent Nrf2 activator, on pAKT is independent of Nrf2. In line with these results, and like the effect of SFN alone (Figure 1B), the combination treatment VVD130037/SFN decreased AKT1 levels in zebrafish larvae (Figure 5A). This result further supports the possibility that SFN potentially acts, at least in part, in an Nrf2-independent manner to reduce AKT1 levels, which we observed in a zebrafish model of glioblastoma initiation. Further investigations in a mammalian model of glioblastoma are required to elucidate the basis of SFN and HBB2 action on AKT1 and pAKT1 accordingly.

### 3.4. Combining Treatment with SFN and VVD130037 with Microglia/Macrophage Elimination Leads to a Further Reduction of the Levels of AKT1

A previous publication from our group demonstrated microglia/macrophage-regulated proliferation of AKT1 glioblastoma-initiating cells [25]. Overexpressing AKT1 in irf8-/- mutant larvae, which lack a microglia/macrophage population, resulted in a significant decrease in proliferating neural progenitors expressing AKT1, compared to the proliferation rate in AKT1-positive cells in wild-type larvae [25]. Based on these data, we aimed to assess the effectiveness of combining microglia/macrophage elimination with SFN and VVD130037 treatment on the AKT1 levels. In this experiment, we solely focused on obtaining a protein sample from the embryo head. We collected protein samples from the following experimental groups: AKT1 overexpressing wild-type (WT) larvae treated with vehicle, IRF8-/- larvae treated with vehicle, and IRF8-/- larvae treated with a combination of VVD130037 and SFN. In line with previous work [25], we observed a decrease in AKT1 levels in IRF8-/- larvae compared with WT, but interestingly, the decrease was more pronounced in IRF8-/- mutant larvae treated with the combination of VVD130037/SFN (Figure 5B).

## 4. Discussion

In search of complementary cancer therapies, research into bioactive compounds found in everyday diets has increased in recent years [26]. SFN, one of the most potent naturally occurring Nrf2 inducers, has demonstrated multifaceted anti-tumorigenic activity and the ability to cross the blood-brain barrier [27,28,29]. However, high Nrf2 levels are associated with increased angiogenesis, a greater capacity for malignant cells to evade immune cells, and increased antioxidant defenses facilitating tumor growth [7]. Thus, administration of an Nrf2 inhibitor should have resulted in an anti-tumorigenic phenotype. Intriguingly, we showed that this was not the case in the AKT1 glioblastoma initiation model in zebrafish. Following treatment of zebrafish larvae with the Nrf2 inhibitor VVD130037, we detected a slight increase in AKT1 protein levels. For these experiments, we set out to obtain two biological replicates for protein quantification from each treatment combination due to low breeding rates of IRF8-/- and fragile larvae, which we recognise is a study limitation and attempted to offset this effect by collecting a pooled protein sample from several wild-type zebrafish larvae with mixed genetic background. Importantly, the VVD130037 larval zebrafish results are in line with our data in human osteosarcoma (U2OS) cells, where Nrf2 deficiency led to an increase in the levels of pAKT. By contrast, exposure to low, but not high, concentrations of SFN in AKT1-overexpressing zebrafish decreased AKT1 protein levels. While preliminary and lacking validation in a mammalian model of glioblastoma, we highlight the importance of careful consideration of both the optimal SFN concentration and Nrf2 activation for achieving beneficial effects.

Differential epigenetic regulation of Nrf2 in different cell populations poses another set of considerations [24]. The failure of SFN to induce Nrf2 activation in neural progenitors and the decrease in AKT1 protein level in response to the combination treatment of VVD130037/SFN suggest an Nrf2-independent anti-tumorigenic action of SFN and the existence of a non-tumorigenic cell population that is sensitive towards changes in Nrf2 activity level. SFN has been shown to increase p53 levels, resulting in caspase-3 activation and apoptosis [10]. Although further work is required for a precise understanding, increased apoptosis is an unlikely reason for the SFN-mediated effects in AKT1-overexpressing neural progenitors, since we did not observe an increase in acridine orange-positive apoptotic preneoplastic cells. SFN has multiple other cellular targets, including Nrf2-independent inhibition of the serine/threonine protein kinase mechanistic target of rapamycin (mTOR) and activation of 5′-AMP-activated protein kinase (AMPK) [19,30]. Further studies are required to identify exactly which mechanism(s) are responsible for the SFN-mediated decrease in AKT1 protein level.

Notably, SFN has also been shown to inhibit the activity of histone deacetylases, including cytoplasmic histone deacetylase 6 (HDAC6) [31,32]. This could result in further downregulation of the kinase activity of AKT, as we have previously observed in another context [33]. The inhibition of HDAC6 by SFN could be particularly relevant to AKT-driven glioblastoma, as it has been shown in human neural progenitor cells that inhibition of HDAC6 increases the acetylation of K163 and K377 in the AKT kinase domain, decreasing the enzyme activity of AKT [33]. It is also notable that both Nrf2 activation and inhibition of HDAC activity have been reported in peripheral blood mononuclear cells (PBMC) isolated from human subjects following consumption of SFN-containing broccoli sprouts [32,33].

Inhibition of AKT and a decrease in cell proliferation by SFN have also been observed in AKT-overexpressing murine and human ovarian cancer cell lines as well as in murine adipocytes [34,35]. Interestingly, SFN has been shown to activate Phosphatase and Tensin Homolog (PTEN), which counteracts the activity of AKT. Both the inhibition of HDAC6, which deacetylates and inactivates PTEN, and the suppression of microRNAs, such as miR-19 and miR-21, which target and inhibit PTEN, have been implicated in the mechanisms by which SFN activates this tumor suppressor [36,37,38].

## 5. Conclusions

In our previous work, we showed the dependence of the proliferative activity of AKT1-overexpressing cells on the presence of microglia/macrophages. In this report, we confirm that eliminating microglia/macrophages leads to a decrease in AKT1 levels. Moreover, our current results further suggest that SFN, in combination with the Nrf2 inhibitor VVD130037, has the potential to decrease AKT1 levels. These results highlight the need to approach glioblastoma therapy as a multifaceted problem. Future single-cell resolution studies are necessary to reveal which functional pathways are activated in different cell populations during combination treatment with VVD130037/SFN.

## Data Availability

All Data are available in the Supplementary Materials section.

## Supplementary Materials

The following supporting information can be downloaded at: https://www.mdpi.com/article/10.3390/nu18162694/s1.
