# Supplementary material for: Sulforaphane Decreases the Burden of AKT1-Expressing Pre-Neoplastic Cells in a Zebrafish Model of Glioblastoma Initiation
Source: Nutrients. 2026 Aug 18;18(16):2694. doi: 10.3390/nu18162694 (PMC13516386; doi:10.3390/nu18162694)
Supplement: Supplementary file 1 [file nutrients-18-02694-s001.zip › nutrients-4483230-supplementary.pdf]

Reagent List

Reagent Type	Designation/ Additional Information	Corresponding Figure
Antibody	ant-4c4(mouse monoclonal, Sieger Laboratory, University of Edinburgh)	Fig2;Fig3
Antibody	Alexa Fluor™ 647(Thermofisher) Catalogue A-21244	Fig2;Fig3
Antibody	ant-AKT1(Invitrogen);Catalog # PA5-29169;Dilution for WB 1:1000	Fig5;Fig1
Antibody	Peroxidase AffiniPure Anti-Rabbit IgG (H+L)(Jackson ImmunoResearch); Catalog#111-035-144;Dilution for WB 1:10000	Fig5;Fig1
Antibody	rabbit monoclonal anti-NRF2(CST), 1:1000, CST	Fig6
Antibody	rabbit polyclonal anti-HO-1(Enzo Life Sciences), Dilution for WB1:1000	Fig 6
Antibody	rabbit monoclonal anti-phospho-AKT (S473)(CST), Dilution For WB1: 1000	Fig 6
Antibody	rabbit monoclonal anti-phospho-AKT (T308)(CST), Dilution for WB 1: 1000	Fig 6
Antibody	mouse monoclonal anti-β-actin antibody(Sigma), Dilution for WB1:20000	Fig 6
Protein Ladder	Precision Plus Protein Dual Color Standards, Catalogue #1610374	Fig5;Fig1
Commercial assay or kit	Rneasy Plus Micro kit(QIAGEN);Catalog # 74034	Fig3;Fig2
Commercial assay or kit	Super-Script® III First-Strand Synthesis System (Invitrogen);Catalog# 18080-051	Fig3;Fig2
Commercial assay or kit	SsoAdvanced Universal SYBR Green Supermix (Bio-Rad);Catalog# 1725271	Fig3;Fig2
Commercial assay or kit	Pierce™ BCA Protein Assay Kits(Thermo Scientific™); Catalog# 23227	Fig5;Fig1
Commercial assay or kit	ECL Select™ Western Blotting Detection Reagent(cytiva); Catalog# RPN2235	Fig5;Fig1
Commercial assay or kit	Mini-Protein TGX Precast Gels(Bio-Rad); Catalog# 4561085	Fig5;Fig1
Commercial assay or kit	Immuno-Blot LF PVDF Membrane and Filter papers(Bio-Rad); Catalog# 1620262	Fig5;Fig1
Commercial assay or kit	PrimeScript™ IV 1st strand cDNA Synthesis Mix(Takara Bio)Catalogue #6215A	Fig4
Commercial assay or kit	PureLink™ RNA Mini Kit(Invitrogen™)Catalogue#12183020	Fig4
Commercial assay or kit	PowerUp™ SYBR™ Green Master Mix for qPCR(Applied Biosystems™)CatalogueA25741	Fig4

Chemicals	acridine orange(Sigma Aldrich) Catalogue#235474	Fig1
Chemicals	HBSS 1x(Gibco) Catalogue# 14170-088	Fig1;Fig2;Fig3 Fig5
Chemicals	D-Glucose(Sigma Aldrich) Catalogue # G8644	Fig1;Fig2;Fig3;Fig5
Chemicals	HEPES(Gibco) Catalogue#15630-056	Fig1;Fig2;Fig3;Fig5
Chemicals	2-Mercaptoethanol(Gibco); Catalog # 31350-010	Fig1 Fig5
Chemicals	Tricaine(Sigma Alrdich) Catalogue # A5040	Fig1;Fig2;Fig3;Fig5
Chemicals	DMSO(Sigma) Catalogue # D8418	Fig5
Chemicals	Ethanol	Fig1;Fig2;Fig3;Fig5
Chemicals	D-Sulforaphane(S-Sulforaphane)(LKT Laboratories) Catalogue S8045	Fig2;Fig3
Chemicals	D,L-Sulforaphane(Merc) Catalogue#57421	Fig1;Fig5
Chemicals	VVD130037(TargetMol)Catalogue # 3034880-93-5	Fig5
Chemicals	RIPA(Sigmal Alrdich) Catalogue # R0278	Fig1;Fig5
Chemicals	PI(Protease Inhibitor)(Thermo Scientific) Catalogue# A32953	Fig1;Fig5

Figure 1A Source data
(Calculating Total RFP Volume)

ETH	10μM D,L -Sulf	40μM D,L-Sulf
176361.9	68442.38161	107745.8562
144202.4	102047.4161	26606.10001
70339.35	108147.5391	30748.72691
59038.57	41119.27208	78165.90406
69588.34	12797.91	91729.82221
141601.4	13759.34	40407.12
32938.68	32795.15	61406.62353
97970.94	32865.83765	108627.1127
41474.92	51546.11215	55985.25
56937.97	55145.49921	63764.61987
88516.06	38870.2182	27534.61797

Figure 1B Source data
(Calculating Relative AKT1 Expression)

ETH	10μM D,L -Sulf	40μM D,L-Sulf
1	0.900242	1.118276261
1	0.76707	1.195833627

Figure 1C Source data
(Calculating Percentage of Apoptotic Cells)

ETH	10μM D,L -Sulf	40μM D,L-Sulf
2.937525999	6.34000636	11.0673927
4.87488202	2.896270414	0
3.229270766	1.746528156	0
2.379659613	4.054161039	7.259559343
0.369108168	0	0
14.28086469	1.464401106	0.451649179
0.387448505	3.910179671	1.494793804
2.962822222	0	4.420703125
0	0.331184775	3.293714663
0	0	7.178390901
6.304419767	1.658534019	2.971230062
0		0.374306234

Figure 3 Source Data

(Calculating fold change for gclc expression)

ETH/Microglia Macrophage	40μM Sulf/Microglia Macrophage
1.27308	8.996976
0.84294	6.65118
0.93185	2.327777

ETH/Mixed cell population	40μM Sulf/Mixed cell population
1.17908	1.632029
0.848116	1.678493

Figure 4

(Calculating Nrf2 Target Gene Expression Fold Change)

NFE2L2		
**DMSO**	**500 nM**	**100 nM**
1.144042	1.306073	1.208047
1.123522	1.151168	1.015338
0.777994	1.092271	0.861832

KEAP1		
**DMSO**	**500 nM**	**100 nM**
0.99269	0.865793	0.905376
0.984902	0.878373	0.836115
1.022806	0.904014	1.013944

NQO1		
**DMSO**	**500 nM**	**100 nM**
0.988894	0.384612	0.412289
0.819657	0.391168	0.291032
1.233723	0.816127	0.695451

GCLM		
**DMSO**	**500 nM**	**100 nM**
0.93598	0.615891	0.662348
0.967205	0.659778	0.596713
1.104625	0.746109	0.857474

HMOX1		
**DMSO**	**500 nM**	**100 nM**
0.929727	0.622333	0.646319
0.915781	0.67986	0.64999
1.174501	0.835081	0.779888

Figure 5A Source data
(Calculating Relative AKT1 Expression)

Eth/DMSO WT	VVD130037	VVD130037+10µM D, L-Sulf
1	1.133	0.926
1	1.127	0.856

Figure 5B Source data
(Calculating Relative AKT1 Expression)

Eth/DMSO WT	Eth/DMSO IRF8-/-	IRF8-/- VVD130037+10µM D,L-Sulf
1	0.689383	0.79258
1	0.8500647	0.503578

Uncropped Immonoblot Images

Fig.1B

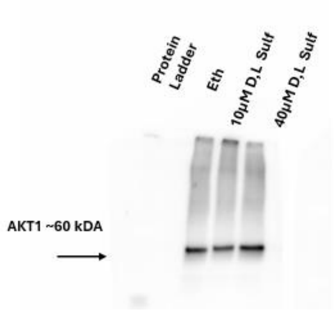

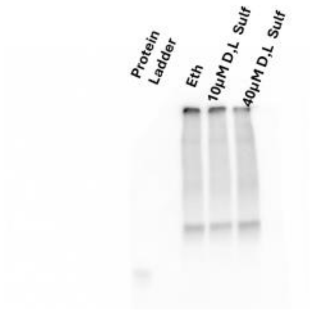

Fig.5A

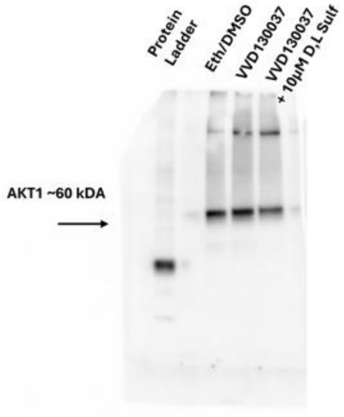

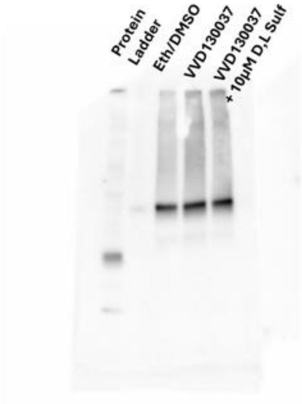

Fig.5B

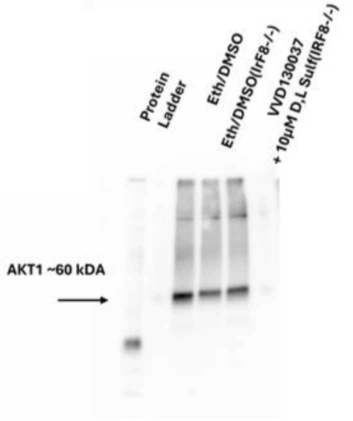

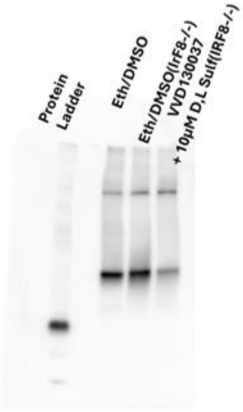

Fig.6

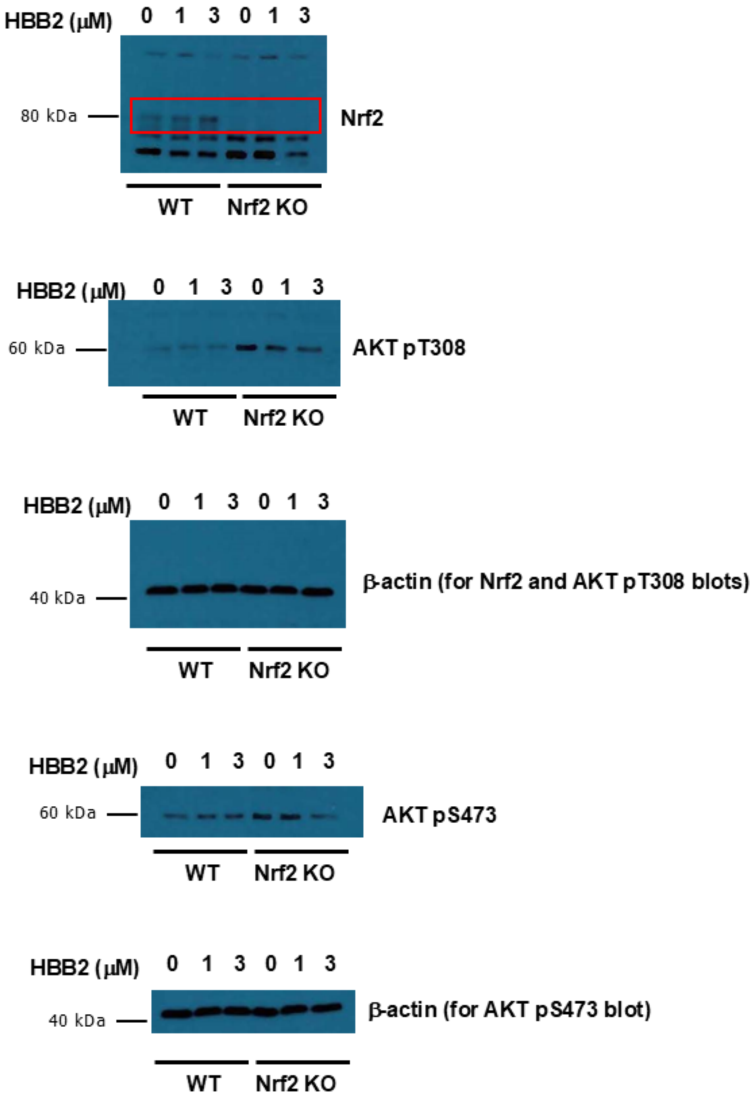
HBB2 (μM) 0 1 3 0 1 3
80 kDa
Nrf2
WT
Nrf2 KO
HBB2 (μM) 0 1 3 0 1 3
60 kDa
AKT pT308
WT
Nrf2 KO
HBB2 (μM) 0 1 3 0 1 3
40 kDa
β-actin (for Nrf2 and AKT pT308 blots)
WT
Nrf2 KO
HBB2 (μM) 0 1 3 0 1 3
60 kDa
AKT pS473
WT
Nrf2 KO
HBB2 (μM) 0 1 3 0 1 3
40 kDa
β-actin (for AKT pS473 blot)
WT
Nrf2 KO
